# Prion 2024 conference: from two decades of growth to a new journey forward

**DOI:** 10.1080/19336896.2025.2514569

**Published:** 2025-06-03

**Authors:** Yifei Kong, Pengcheng Huang, Qi Shi, Yifan Wang, Mengting Li, Jiyan Ma, Xiao-Ping Dong, Daojun Hong, Wen-Quan Zou

**Affiliations:** a Institute of Neurology, Jiangxi Academy of Clinical Medical Sciences, The First Affiliated Hospital, Jiangxi Medical College, Nanchang University, Nanchang, Jiangxi, China; b National Key-Laboratory of Intelligent Tracking and Forecasting for Infectious Disease, National Institute for Viral Disease Control and Prevention, Chinese Center for Disease Control and Prevention, Beijing, China; c Beijing Institute for Brain Research, Chinese Academy of Medical Sciences & Peking Union Medical College, Beijing, China; d Chinese Institute for Brain Research, Beijing, China

**Keywords:** Alzheimer’s disease (AD), biomarkers, chronic wasting disease (CWD), conference, Creutzfeldt-Jakob disease (CJD), epidemiology, Parkinson’s disease (PD), prion diseases, prions, strains, surveillance

## Abstract

The Prion 2024 annual conference, held in Nanchang, China, from 23 to 27 October, drew nearly 300 leading scientists, clinicians, researchers, and students from 17 countries to examine the latest advancements in prion research and related diseases. This landmark event marked the inaugural international prion conference hosted in a developing nation for the first time and celebrated the 20th anniversary of the NeuroPrion Association, the organizing body of this prestigious annual gathering – ‘*Celebrating Two Decades of Progress: Pioneering a New Era*.’ The conference spotlighted key themes such as epidemiology, pathogenesis, the connections between ageing and neurodegenerative diseases. It showcased innovative diagnostic and therapeutic strategies for both human and animal prion diseases, as well as related conditions including Alzheimer’s and Parkinson’s diseases, prion protein-associated cancers, and renal injury. The programme featured 70 invited talks and 21 selected oral presentations, culminating in 7 plenary sessions led by esteemed speakers, including Nobel Laureate Stanley B. Prusiner. This overview summarizes key presentations and highlights significant aspects of the conference, emphasizing the impactful discussions and collaborations that emerged from this historic event.

## Introduction

Prion diseases are a group of fatal, transmissible neurodegenerative disorders that impact both humans and animals [1]. In humans, notable conditions include Creutzfeldt-Jakob disease (CJD), kuru, variant CJD, Gerstmann – Sträussler – Scheinker syndrome (GSS), fatal insomnia (FI), and variably protease-sensitive prionopathies (VPSPr) [2]. Among animals, significant examples include scrapie in sheep and goats, bovine spongiform encephalopathy (BSE, commonly known as mad cow disease) in cattle, and chronic wasting disease (CWD) in deer, elk, and moose [3]. While these diseases are rare, they captured global attention during the BSE crisis of the 1980s and the subsequent transmission to humans in the 1990s in the United Kingdom and Europe, raising substantial public health concerns [4]. This urgency prompted the establishment of the NeuroPrion Association by the European Union in 2004 to coordinate research and responses to prion diseases. The association held its inaugural annual conference in Paris that year and has since organized it annually, with exceptions in 2020 and 2021 due to the COVID-19 pandemic, creating a vital international forum for researchers to exchange advancements and strategies in addressing these complex conditions.

The Prion 2024 annual conference took place in Nanchang, Jiangxi Province, China, from 23 to 27 October 2024. Sponsored by the NeuroPrion Association, Nanchang University, the Chinese Center for Disease Control and Prevention, and the Neurodegenerative Disease Branch of the Chinese Neuroscience Society, the event was hosted by the First Affiliated Hospital of Nanchang University, the Jiangxi Society of Integrative Medicine, and the National Key Laboratory of Infectious Disease Traceability, Early Warning, and Intelligent Decision-making. This conference marked a significant milestone as the first hosted in a developing country and the second in Asia. The scientific committee organized 70 invited talks, including 7 plenary speeches, covering crucial topics such as epidemiology, surveillance, pathogenesis, diagnosis, and treatment of both human and animal prion diseases, along with related disorders like Alzheimer’s disease (AD), Parkinson’s disease (PD), amyotrophic lateral sclerosis (ALS), and neuronal intranuclear inclusion disease (NIID). It has been proposed that the misfolded proteins in these conditions share a similar prion-like mechanism for propagation [5]. In addition, the conference featured 21 oral presentations by emerging scholars and recognized excellence with awards for the best posters presented by 16 graduate students and postdoctoral fellows.

Attracting nearly 300 participants from 17 countries, including Australia, Brazil, Canada, China, France, Germany, India, Italy, Japan, New Zealand, Pakistan, Russia, South Korea, Spain, Switzerland, the UK, and the USA, Prion 2024 coincided with the 20th anniversary of the NeuroPrion Association and the annual conference. This occasion celebrated two decades of milestones while welcoming a new chapter in prion research. The agenda emphasized critical academic issues such as the transmission and pathogenesis of prion diseases, clinical diagnostic techniques, treatment strategies, and global public health policies. Sessions included titles like ‘Prion Strains: From Molecular Structures to Disease Diversity,’ ‘Advances in Pathogenic and Diagnostic Studies,’ and ‘Advances in Therapeutic Studies,’ encompassing a total of 10 primary topics and 3 workshops. This overview aims to summarize key presentations and highlight significant aspects of the conference presented and published in the *Prion* journal, offering insights into various aspects of prion and prion-related diseases [6].

## Key themes and topics

The conference featured 10 main tracks: Epidemiology/surveillance, Pathogenic mechanisms, Diagnostic biomarkers, Prion-like diseases, Prion strains: from molecular structures to disease diversity, Ageing and neurodegenerative diseases, Animal prion diseases, CJD international support alliance, Glial cells in neurodegenerative diseases, and Hot topics.

### Epidemiology/surveillance

Robust epidemiological data are essential for comprehending prion diseases and addressing public health concerns. Cutfield et al. highlight 25 years of monitoring CJD in New Zealand, revealing consistent incidence rates with a particular emphasis on sporadic cases. In parallel, Tsukamoto et al. provide insights into the epidemiology of prion diseases in Japan, noting a rise in reported cases, which underscores the necessity for ongoing surveillance to monitor genetic mutations and varying disease types. Maddox et al. mortality surveillance of CWD exposure emphasizes that no human prion disease cases have been linked to the consumption of infected cervids; however, continued monitoring remains crucial due to the lengthy incubation period associated with CWD. Shi et al. report on the characteristics of human prion diseases in China, based on two decades of surveillance data, identifying genetic CJD as the most common mutation, specifically PrP T188K or E200K, along with FFI linked to D178N mutations. Stehmann *et al*. also presented updates on Australian surveillance of human prion diseases, indicating that 92.5% of cases are sporadic CJD, with genetic and iatrogenic cases accounting for 8.5% and <1%, respectively. Notably, no cases of variant CJD have been reported, while the first case of VPSPr was identified through ongoing ANCJDR PrP^Sc^ glycotyping.

### Pathogenesis

While it is well-established that PrP^Sc^ is the primary molecule involved in the pathogenesis of various prion diseases, the exact molecular mechanisms underlying the conversion of PrP^C^ to PrP^Sc^ and the resulting neuronal injury remain inadequately defined. Studies on the pathogenesis of prion diseases revealed diverse mechanisms influencing prion propagation, strain variability, and neurodegeneration. Adriano Aguzzi introduced their recent studies of the prion life cycle through genetic screens, identifying 80 up- and 451 down-regulators of PrP^C^ expression. Many of these were non-canonical transcription factors impacting various cellular functions. The study linked extracellular matrix (ECM) organization to PrP^C^ levels, associating gliotic tissue stiffness with prion disease. Also, they observed that the Bone Morphogenetic Protein (BMP) pathway was crucial for prion internalization, while ciliogenesis contributed to prion toxicity. Claudio Soto presented the prion principle, detailing how misfolded proteins, like those involved in prion diseases, AD and PD, propagate. He highlighted the evolution of prion research and the importance of amplifying misfolded proteins to understand disease mechanisms. Advances such as cryogenic electron microscopy and single-cell RNA sequencing are emphasized as valuable tools for diagnosis and research into neurodegenerative diseases. Alam *et al*. identified distinct structural conformations of infectious prion fibrils that affect strain propagation. Hill *et al*. highlighted the role of Syntaxin-6 in moderating prion infection dynamics in sCJD. Shi et al. discovered unstable CWD strains in Scandinavian cervids, emphasizing the significance of strain diversity. Additionally, Iwasaki et al. linked tube feeding to longer survival in gCJD patients. Through various methods, studies by Vanni *et al*. and Xiong focused on the infectious potential of oligomeric PrP^Sc^ aggregates and the impact of VPS35 deficiency on neuronal pathways, respectively. *In vitro* work revealed that the PrP functions in demyelination through neuroinflammatory processes (Li et al.) and interacts with other proteins, such as α-synuclein and tau (Outeiro and Vieira; Zhan *et al*.), influencing neurodegenerative mechanisms. Furthermore, de Cecco highlighted the significance of BMP signalling in prion uptake, while advances in imaging techniques provided insights into prion propagation dynamics (Trainer *et al*.). Pierluigi Gambetti, through a recorded plenary talk presented by his colleague Laura Cracco, discussed our current understanding of FFI, molecular typing of PrP^Sc^ in human prion diseases, and a novel prion disease, VPSPr. He also presented their recent study demonstrating that PrP glycans reduced spongiform degeneration and PrP^Sc^ deposition in mice. The presence of glycans on PrP significantly influenced the progression of prion diseases. Transgenic mice lacking PrP glycans exhibited higher attack rates and severe histopathology when exposed to prion isolates. These findings underscored the protective role of PrP glycans and revealed the complex conformational changes that occur in PrP^Sc^ following glycan knockout. Altogether, these studies contribute to a nuanced understanding of the complex interplay of genetic, molecular, and environmental factors in prion disease and prion-related pathogenesis.

Regarding other neurodegenerative diseases, the plenary talk by Michel Goedert reviewed the cryo-EM studies of amyloid filament assembly of tau, αSyn, TDP-43, and Aβ, which is central to neurodegenerative diseases. Advances in cryo-EM have elucidated amyloid structures from human brains. His review noted distinct protein folds associated with specific diseases, particularly the critical assembly of Aβ42 in AD. Cryo-EM revealed protofilament types linked to sporadic and familial cases, while tau structures showed minimal variation, informing diagnostics and therapies. Maria Grazia Spillantini also gave a plenary talk to review the discovery, structure and role of αSyn in PD and other synucleinopathies. Vitras mice expressing truncated human αSyn showed that non-aggregated αSyn can spread from the gut to the brain. A 21-nucleotide duplication in SNCA caused juvenile-onset synucleinopathy, introducing a new protein fold. This study indicated that endogenous αSyn aided dopaminergic neuron development without causing cell death, which may initiate pathology in α-synucleinopathies, highlighting the need for therapies targeting αSyn aggregation to mitigate PD.

### Prion structure

Studies on prion structure presented in the conference have revealed significant insights into the conformational diversity and propagation mechanisms of prions. Byron Caughey presented a plenary talk on self-propagating protein seeds as pathogens and biomarkers. His team solved the CWD fibrils’ structure from a white-tailed deer at 2.8 Å resolution, representing the first natural prion structure from a mammalian host with wildtype PrP. These CWD fibrils display a unique structural twist that differentiates them from rodent prions, potentially aiding CWD management. Moreover, simulations of smaller oligomeric fragments of infectious aRML prion fibril cores showed that while dimers quickly lost integrity, larger trimers and octamers maintained stability. Their findings challenge previous notions about prion dynamics and suggest that stable short fragments may contribute to bioactive species in proteinopathy-affected brains. Alam *et al*. examined high-resolution structures of infectious prion fibrils from the brain of prion-infected mouse and deer, uncovering distinct conformations that influence strain propagation and therapeutic approaches. Kulichikhin *et al*. highlighted the structural properties of yeast prion proteins, illustrating how specific regions can maintain prion states despite lacking cross-seeding ability. Wang *et al*. showcased variability in the fibril morphology of the yeast prion [PSI+] based on growth conditions, contributing to the understanding of prion maturation. Trainer *et al*. employed novel imaging techniques to visualize prion strain propagation within neuronal cells, enhancing understanding of their structural dynamics. Vanni *et al*. identified oligomeric PrP^Sc^ aggregates as potentially the most infectious prion particles, retaining strain characteristics without traditional fibril formations. Requena emphasized the active role of PrP^C^ in its conversion to PrP^Sc^, shedding light on fundamental propagation mechanisms. Ernst *et al*. differentiated classical scrapie and CWD strains, illustrating geographical clustering and structural variances affecting zoonotic transmission potential, while Kim *et al*. presented findings on the anti-prion properties of Curcuma phaeocaulis extract, indicating its capacity to inhibit prion aggregation. Collectively, these studies enhance the understanding of prion structures and their implications for pathology and therapeutic strategies.

### Prion strains

Recent studies presented at the conference have advanced our understanding of prion strains and their implications. Alam *et al*. utilized purification and imaging techniques to reveal distinct structural architectures of infectious prion fibrils, influencing strain propagation and therapeutic strategies. Shi *et al*. documented the emergence of unstable CWD strains in Nordic cervids, emphasizing strain diversity and adaptation during transmission. Vanni *et al*. demonstrated the infectious potential of oligomeric PrP^Sc^ aggregates, suggesting these may be more effective propagators of prion diseases than traditional fibrils. DeFranco *et al*. assessed the influence of transmission routes on CWD strain propagation, revealing that peripheral and intracerebral challenges produce distinct prion strains with varying biochemical properties. Additionally, Lang et al. identified a unique PrP^Sc^ species linked to gCJD detected mainly by antibodies against C-terminal PrP fragments but not by the most widely used 3F4 antibody, contributing to our understanding of genetic prion disease mechanisms. Collectively, these studies underscore the complexity and evolving nature of prion strains, highlighting their diverse impacts on pathology and potential therapeutic targets.

### Aging and neurodegenerative diseases

The conference abstracts presented various studies exploring the relationship between ageing and neurodegenerative diseases, particularly prion diseases and synucleinopathies. The presentation by Jean-Philippe Deslys focused on the correlation between ageing and neurodegenerative diseases. He pointed out that the ageing global population is expected to double by 2050, increasing diseases linked to protein aggregation, especially in brain tissues. Prion mechanisms are thought to contribute to neurodegenerative progression. Studies on natural models, including mice and primates, have revealed atypical prion strains that evade standard diagnostics, suggesting broader implications of prion-like phenomena in ageing. Tian et al. identified age-shifted vascular genes through bulk RNA sequencing from human and mouse vessels. They revealed conserved transcriptional changes linked to vascular ageing that affect extracellular matrix organization, metabolism, and inflammation, contributing to atherosclerotic diseases. Age-related shifts in gene expression increased susceptibility to complex diseases in the elderly.

### New prion concepts

Stanley Prusiner’s plenary speech expanded the prion paradigm to encompass proteinopathies such as AD and PD emphasizing conformational diversity as a unifying characteristic. Recent research has well-demonstrated that Aβ and tau from AD and αSyn from PD are of prion-like transmissibility. In light of these findings, Prusiner argued that all should be classified as prions, rendering the search for alternative terms or ambiguous labels like ‘prion-like’ unnecessary. Alam et al. resolved high-resolution structures of prion fibrils, revealing strain-specific conformations associated with infectivity, while Vanni et al. identified oligomeric PrP^Sc^ aggregates – rather than fibrils – as the most infectious particles, retaining strain properties and propagating disease. Trainer et al. developed real-time imaging techniques to track prion dynamics in living cells, uncovering structural transitions during propagation. Further studies revealed prion-like mechanisms in non-prion proteins: Belousov et al. demonstrated amyloid-forming bacterial porins, suggesting evolutionary parallels in pathogenic protein aggregation, while Zhan et al. showed cross-seeding interactions between αSyn and tau, driving co-aggregation in neurodegenerative diseases. Requena redefined the active role of PrP^C^ in its own pathogenic conversion, and De Cecco et al. identified BMP signalling as a novel pathway for prion cellular uptake. Furthermore, therapeutic strategies rooted in structural insights were presented, including lipid-based siRNA delivery systems (Bender et al.) and conformation-targeting vaccines (Sriraman et al.). Collectively, these studies redefine prions as dynamic, structurally plastic entities with broader implications for neurodegeneration, host–pathogen interactions, and systemic disease, emphasizing the need for updated diagnostic and therapeutic frameworks.

### Diagnostic biomarkers

Recent advancements in prion disease diagnostics emphasize non-invasive, sensitive, and early detection methods. Correia *et al*. developed a tear fluid RT-QuIC assay, demonstrating high sensitivity for sporadic and genetic prion diseases, while Alvarado *et al*. utilized flow cytometry to measure PrP levels in blood, enabling real-time monitoring of therapeutics. Nonaka *et al*. highlighted challenges in blood-based detection but noted improved sensitivity with the eQuIC assay, despite remaining limitations. Favole *et al*. identified prion secretion in milk from scrapie-infected sheep using RT-QuIC, offering a novel surveillance route. Beyond prions, Yang *et al*. adapted RT-QuIC to detect tau seeding activity in blood for Alzheimer’s diagnosis, reflecting cross-disease applicability. Skin biopsies emerged as a promising tool: Zou *et al*. detected misfolded prions, α-synuclein, and tau via RT-QuIC, while Donadio *et al*. leveraged phosphorylated α-synuclein in skin samples to differentiate Parkinson’s from other disorders. Imaging biomarkers also gained traction – Jing and Wu revealed early white matter integrity changes in familial CJD carriers via diffusion tensor imaging. Cheng *et al*. enhanced CSF diagnostics by integrating the 14–3–3 protein into RT-QuIC, improving reproducibility. Unconventional biomarkers, such as unexpected weight gain in sCJD (Ellett *et al*.) and renal PrP^C^ interactions (Chen *et al*.), broadened diagnostic horizons. Orru *et al*. highlighted advancements in detecting misfolded proteins in various biospecimens using RT-QuIC assays. The findings demonstrate the potential for early diagnosis of neurodegenerative diseases based on peripheral biomarker accumulation. Kuang *et al*. from the Xu lab described a novel seed-amplification assay (SAA) termed quiescent SAA (QSAA) that was able to amplify misfolded α-Synuclein in tissue lysates and tissue sections. Together, these studies underscore a shift towards peripheral, non-invasive biomarkers and multisystem approaches, enhancing early detection and disease-specific differentiation across neurodegenerative conditions.

### Treatment and prevention

Recent advances in prion disease therapeutics and prevention highlight innovative strategies targeting protein misfolding and propagation. Therapeutic approaches include small molecules (Situ *et al*.) that block prion protein secretion, antibodies (Song *et al*.) inducing PrP degradation, and PrP^C^ antagonists like PSCMA (Wang *et al*.), which prolonged survival in murine models. Natural compounds such as Curcuma phaeocaulis extract (Kim *et al*.) and Moringa oleifera derivatives (Amaral-do-Nascimento *et al*.) showed promise in inhibiting prion aggregation. Vaccines have emerged as key tools: structure-based designs targeting amyloid beta and tau (Sriraman *et al*.), α-synuclein-mimicking fibrils for Parkinson’s (Ma *et al*.), and fungal prion-based vaccines for CWD in wildlife (Fang *et al*., Schatzl *et al*.). Gene-editing technologies, including CRISPR screens (De Cecco *et al*.), identified BMP signalling as a prion uptake pathway, while siRNA delivery via liposomes (Bender *et al*.) effectively reduces PrP levels. Novel peptides (Willbold *et al*.) stabilize monomeric proteins, and plasmin-mediated cleavage (Ryou *et al*.) offers anti-prion potential.

Preventive strategies focus on surveillance and environmental controls. CWD monitoring via milk (Favole *et al*.) and non-invasive diagnostics (e.g., tear fluid RT-QuIC) aid early detection. Environmental interventions, such as copper application to degrade prions (Yuan *et al*.), and genetic resistance studies in goats (Elosua *et al*.) inform management practices. Global surveillance programmes in China, Australia, and New Zealand (Shi, Stehmann, Cutfield *et al*.) underscore the importance of robust public health frameworks. These studies reflect a multidisciplinary push towards targeted therapies, structural biology-driven interventions, and proactive prevention to mitigate prion and related neurodegenerative diseases.

## Key findings and insights

The conference collectively advanced our understanding of prion diseases and related neurodegenerative disorders through structural, therapeutic, diagnostic, and epidemiological insights. Structural studies revealed that prion strain diversity (Alam *et al*.) and oligomeric PrP^Sc^ aggregates (Vanni *et al*.) drive infectivity, while bacterial porins (Belousov *et al*.) and yeast prion-phase separation (Gorsheneva *et al*.) underscore broader protein aggregation mechanisms. Diagnostic innovations include non-invasive methods such as tear fluid RT-QuIC (Correia *et al*.), skin biopsy detection of misfolded proteins (Zou *et al*.), and blood-based tau seeding assays for Alzheimer’s (Yang *et al*.), alongside white matter imaging in preclinical CJD (Jing *et al*.). Therapeutic strategies featured small molecules (Situ *et al*.), antibodies (Song *et al*.), and PrP^C^ antagonists (Wang *et al*.) to reduce prion propagation, while vaccines targeting α-synuclein (Ma *et al*.), CWD (Fang *et al*.), and amyloid beta/tau (Sriraman *et al*.) showed promise. Natural compounds like Curcuma (Kim *et al*.) and Moringa (Amaral-do-Nascimento *et al*.) inhibited aggregation, and gene-editing tools (Bender *et al*.’s siRNA liposomes, De Cecco *et al*.’s CRISPR screens) identified novel targets. Pathogenesis insights highlighted Syntaxin-6’s role in toxic aggregation (Sangar *et al*.), microglia-astrocyte interactions (Mabbott *et al*.), and plasmin-mediated cleavage (Ryou *et al*.). Cross-disease links emerged, such as α-synuclein-tau co-aggregation (Zhan *et al*.), Aβ deposits in VPSPr-infected primates (Comoy *et al*.), and mitochondrial dysfunction via SIRT1 (Zhao *et al*.). Surveillance and prevention emphasized CWD’s zoonotic risk (Shi, Barrio *et al*.), environmental prion degradation using copper (Yuan *et al*.), and global monitoring programmes (China, Australia, New Zealand). The expanding prion paradigm (Prusiner) now encompasses Alzheimer’s and Parkinson’s, driven by conformational plasticity and shared mechanisms like proteopathic seeding and transmissibility. Interestingly, the role of PrP^C^ extends to renal injury as well. Nie et al. investigated the involvement of PrP^C^ in renal fibrosis (RF) and found that elevated levels of PrP^C^ were present in fibrotic renal tissues from both mice and patients with chronic kidney disease. The absence of PrP^C^ mitigated RF following kidney injury by activating profibrotic responses via the TBK1-IRF3 pathway. Furthermore, inhibiting TBK1 reduced RF, suggesting that the PrP^C^-TBK1-IRF3 axis could serve as a promising therapeutic target and indicating the significance of biomolecular condensation in organ fibrosis. Contrastingly, Song et al. explored the regulation of RF by PrP^C^ following kidney injury, utilizing both wild-type and Prnp-/- mice. Renal injury was induced through ischaemia-reperfusion and unilateral ureteral obstruction. They found that increased levels of PrP^C^ in injured renal proximal tubules were associated with exacerbated injury in PrP^C^-deficient mice, indicating activation of the EGFR pathway. Also, elevated urinary PrP^C^ levels in patients suggest its potential as a non-invasive biomarker for renal injury, further highlighting its role in renal repair.

## Challenges

The conference highlighted several challenges faced in the study and treatment of prion diseases and related neurodegenerative disorders. A primary concern is the emergence of unstable CWD strains, as reported by Shi *et al*., which raises issues of strain diversity and the potential for zoonotic transmission. Detection of prion activity remains problematic, with Nonaka *et al*. noting the limitations of current methods for identifying prion seeding in blood and the need for more reliable alternatives. The variability in genetic factors, such as those affecting prion transmission in goats (Elosua *et al*.), underscores the complexity involved in understanding and managing these diseases. Mabbott *et al*. also highlighted how microglial responses can impact disease progression, indicating a need for a deeper understanding of host immune responses. The challenges posed by neuroinflammation and its effects on prion pathology, combined with the difficulty of detecting early signs of disease, call for advancements in diagnostic and therapeutic strategies. Furthermore, the intricate interactions between proteins like α-synuclein and tau, as explored by Zhan *et al*., emphasized the difficulties in unravelling the underlying mechanisms driving neurodegeneration. These challenges necessitate ongoing research and innovative approaches to effectively address the complexities of prion-related diseases and improve patient outcomes.

## Future directions

The conference indicates several future research directions aimed at advancing our understanding and treatment of prion diseases and neurodegenerative disorders. Future studies should focus on elucidating the mechanisms underlying prion strain propagation and the specific roles of associated proteins, such as Syntaxin-6, in disease pathogenesis. The identification of new therapeutic strategies, including small-molecule inhibitors and PrP-targeting antibodies, should be prioritized to enhance PrP management. Investigating the zoonotic potential of emerging CWD strains, particularly in light of their adaptability and interspecies transmission, is crucial for public health considerations. Moreover, leveraging advanced diagnostic techniques, including RT-QuIC and QSAA assays for early detection in various biological samples, could significantly improve diagnostic accuracy. The development of vaccines based on structural biology to combat misfolded proteins, alongside ongoing genetic studies to identify susceptibility factors, promises to inform future interventions. Finally, enhancing our understanding of neuroinflammation’s dual roles, the interaction between α-synuclein and tau proteins, and employing novel gene manipulation techniques through CRISPR could provide pivotal insights for therapeutic development in neurodegenerative diseases. All these future directions emphasize the integration of molecular biology, immunology, and innovative diagnostic approaches to tackle the complexities of prion-related diseases and associated neurodegenerative conditions.

## Looking forward to prion 2025

Prion 2025 conference will take place in Búzios, Rio de Janeiro, Brazil, from 3 to 7 November 2025, for which, more detailed information can be found at https://eventos.galoa.com.br/prion-2025/page/5178-home.

## Data Availability

The data that support the findings of this study are available on request from the corresponding author, **WQZ**. The data are not publicly available due to restrictions *e.g*. their containing information that could compromise the privacy of research participants.
